# Limited DNA methylation variation and the transcription of *MET1* and *DDM1* in the genus *Chrysanthemum* (Asteraceae): following the track of polyploidy

**DOI:** 10.3389/fpls.2015.00668

**Published:** 2015-08-27

**Authors:** Haibin Wang, Xiangyu Qi, Sumei Chen, Weimin Fang, Zhiyong Guan, Nianjun Teng, Yuan Liao, Jiafu Jiang, Fadi Chen

**Affiliations:** ^1^College of Horticulture, Nanjing Agricultural UniversityNanjing, China; ^2^Jiangsu Province Engineering Lab for Modern Facility Agriculture Technology and EquipmentNanjing, China

**Keywords:** DNA methylation, *MET1*, *DDM1*, polyploid, *Chrysanthemum*

## Abstract

Polyploidy has been recognized as a widespread and common phenomenon among flowering plants. DNA-5′-CCGG site cytosine methylation (*C*-methylation) is one of the major and immediate epigenetic responses of the plant genome. Elucidating the ways in which altered *C*-methylation patterns, either at the whole genomic level or at specific sites can affect genome stability in polyploidy will require substantial additional investigation. Methylation sensitive amplification polymorphism profiling was used to evaluate variation in *C*-methylation among a set of 20 *Chrysanthemum* species and their close relatives of varying ploidy levels from diploid to decaploid. The range in relative *C*-methylation level was within 10%, and there was no significant difference neither between different ploidy levels nor between different species in the same ploidy level (*U*-values < 1.96). The transcript abundances of *MET1* and *DDM1* genes, which both involved in the regulation of *C*-methylation at CpG sites, were enhanced with increased ploidy level, but only *MET1* was positively correlated with the nuclear DNA content. Considering the key role and efficiency of MET1 in maintaining CpG methylation, the limited variation observed with respect to *C*-methylation may reflect a balance between the increased activity of *MET1* in the higher ploidy genomes and the larger number of CpG dinucleotide sites available for methylation.

## Introduction

Polyploidy is widely recognized as a significant driver of higher plant evolution (Chen, 2007). The majority of extant angiosperm species have undergone at least one whole genome duplication (WGD) event to form either an auto- or an allopolyploid (Jiao et al., 2011). Polyploidization induces changes in both the genome sequence and the transcriptome. At least some of the latter are induced by alterations in cytosine (C-) methylation (DNA-5′-CCGG sites; Salmon et al., 2005; Parisod et al., 2010), a process which underlies much of the epigenetic variation in eukaryotic genomes. Actively transcribed sequences tend to be less heavily methylated than non-active ones, especially in their promoter region (Chan et al., 2005). The phenotypic consequences of altered *C*-methylation patterns can also give rise to evolutionary opportunities (Rangwala and Richards, 2004; Rapp and Wendel, 2005), particularly in the context of polyploidization events (Chen and Ni, 2006; Chen, 2007).

*C*-methylation in higher plant genomes is concentrated within CpG dinucleotides (Lister et al., 2008), and is strongly influenced by the activity of *MET1*, a gene encoding a DNA cytosine-5-methyltransferase, since the suppression of this gene results in a reduction in global *C*-methylation, particularly at CpG sites (Saze et al., 2003). A second important gene in this context is *DDM1* (decrease in DNA methylation), which encodes a likely SNF2/SWI2 class chromatin remodeling protein (Jeddeloh et al., 1999). The functions of DDM1 and MET1 are both involved in the regulation of *C*-methylation at CpG sites (Wang et al., 2004).

The direct detection of *C*-methylation requires a modified form of sequencing (Martienssen and Colot, 2001; Riddle and Richards, 2002; Leitch and Leitch, 2008; Koh et al., 2010), but an indirect and more convenient means is provided by the MSAP (methylation sensitive amplification polymorphism) technique, which exploits the differential sensitivity shown by certain pairs of isoschizomeric restriction enzymes (REs) to *C*-methylation in their recognition site (DNA-5′-CCGG). MSAP based on the enzyme pair *Hpa*II and *Msp*I discriminates between hemi- (^m^CCGG) and fully methylated (C^m^CGG) sites (McClelland et al., 1994; Sha et al., 2005), and has been used to explore the variation in DNA methylation in a number of plant species (Zhao et al., 2007; Wang et al., 2009, 2014b), as well as to demonstrate *C*-methylation induced by polyploidization (Liu et al., 2001; Shaked et al., 2001; Qi et al., 2010).

The Asteraceae genus *Chrysanthemum* includes several polyploid species (Liu et al., 2012; Wang et al., 2013b, 2014c). Considerable variation at the ploidy level is present in this genus (from 2n = 2x = 18, to 2n = 36, 54, 72, up to 90; Liu et al., 2012). In previous studies, we investigated the genomic and epigenomic alterations during intergeneric hybridization in *Chrysanthemum* sp. using MSAP method. Surprisingly, in genus *Chrysanthemum*, the global DNA methylation concentration in the diploids (51.9–53.1%) was not much lower than that in the decaploid (55.4%; Wang et al., 2013a, 2014a,b). The aim of the present study was to characterize species-to-species variation for *C*-methylation in this genus, based on MSAP profiling. In addition, given the key role of MET1 and DDM1 in maintaining CpG methylation (Chen et al., 2010), an analysis of their transcript abundance was undertaken in *Chrysanthemum* species varying in ploidy level from diploid to decaploid.

## Materials and Methods

### Plant Material and DNA Extraction

The plants sampled for DNA analysis have been maintained by vegetative reproduction for at least 8 years under a constant environment (22°C during the day, a minimum at 15°C at night, a relative humidity of 70–75% and under natural light) at the Chrysanthemum Germplasm Resource Preserving Centre, Nanjing Agricultural University, China. The diploids selected were *Chrysanthemum nankingense, C. dichrum, C. japonicum, C. boreale, C. lavandulifolium*, and *Tanacetum vulgare;* the tetraploids were *C. indicum, C. yoshinaganthum, C. okiense, C. japonicum* var. *wakasaense* and *C. chanetii*; the hexaploids were *C. vestitum, C. morifolium, C. japonense*, and *C. zawadskii*; the octoploids were *C. ornatum, Ajania shiwogiku*, and *A. × marginatum*; and the decaploids were *C. crassum* and *A. pacificum* (**Table 1**). Their DNA was extracted from fully expanded fourth and fifth leaves harvested from three plants per species, using a modified CTAB method (Stewart and Via, 1993). DNA integrity was confirmed by running a 2% agarose gel. The concentration and purity of the DNA preparations were monitored using the Nano-Drop ND-1000 Spectrophotometer (Nano-Drop Technologies, Wilmington, DE, USA), and preparations were stored at -20°C for subsequent analysis.

**Table 1:** Taxa used as plant materials and their ploidy level.

Taxa	Ploidy	Reference
*Chrysanthemum boreale*	2x	Watanabe (1983), Wang et al. (2014c)
*C. dichrum*	2x	Zhao et al. (2009), Wang et al. (2014c)
*C. japonicum*	2x	Abd El-Twab and Kondo (2006), Bao et al. (2012)
*C. lavandulifolium*	2x	Zhao et al. (2009), Wang et al. (2014c)
*C. nankingense*	2x	Zhao et al. (2009), Wang et al. (2013a)
*Tanacetum vulgare*	2x	Tang et al. (2011), Wang et al. (2013a)
*C. chanetii*	4x	Abd E-Twab and Kondo (2007), Zhao et al. (2009)
*C. indicum*	4x	Zhao et al. (2009), Tang et al. (2012)
*C. japonicum* var. *wakasaense*	4x	Bao et al. (2012), Wang et al. (2014c)
*C. okiense*	4x	Uehara et al. (2012), Wang et al. (2014c)
*C. yoshinaganthum*	4x	Abd El-Twab and Kondo (2012), Wang et al. (2014c)
*C. japonense*	6x	Watanabe (1983), Wang et al. (2014c)
*C. morifolium*	6x	Zhao et al. (2009), Wang et al. (2014c)
*C. vestitum*	6x	Zhao et al. (2009), Wang et al. (2014c)
*C. zawadskii*	6x	Zhao et al. (2009), Wang et al. (2014c)
*Ajania × marginatum*	8x	Chen et al. (2008), Wang et al. (2014c)
*A. shiwogiku*	8x	Uehara et al. (2012), Wang et al. (2014c)
*C. ornatum*	8x	Watanabe (1981), Wang et al. (2014c)
*C. crassum*	10x	Tang et al. (2009), Wang et al. (2013a)
*A. pacificum*	10x	Uehara et al. (2012), Zhao et al. (2012)

### MSAP Profiling

The interpretation of MSAP data is predominantly based on known RE activities at recognition sequences modified by methylation. Data concerning the methylation sensitivity of REs and corresponding literature can be found at the website of The Restriction Enzyme Database (REBASE^1^) (Fulneček and Kovařík, 2014). The MSAP profiling procedure was based on the protocol given in (Reyna-Lopez et al., 1997; Xiong et al., 1999). About 500 ng DNA per entry was double-digested at 37°C for 12 h in parallel with either 10 U *Eco*RI (New England Biolabs, China, EC 3.1.23.13) and 20 U *Hpa*II (NEB, EC 3.1.23.24), or 10 U *Eco*RI, and 10 U *Msp*I (NEB, EC 3.1.23.24). The products were ligated with 5 pmol *Eco*RI adaptor and 50 pmol *Hpa*II-*Msp*I adaptor (sequences given in Supplementary Table S1) in a reaction containing 4 U T4 DNA ligase held at 16°C for 4 h, after which the ligation reaction was heat-inactivated (65°C, 10 min). A 5 μL aliquot of the product was pre-amplified in the presence of 0.2 μM *Eco*RI and 0.2 μM *Hpa*II-*Msp*I non-selective primers (sequences given in Supplementary Table S1) in a 25 μL reaction containing 2.5 μL 10x PCR buffer, 1.5 mM MgCl_2_, 0.2 mM dNTP, and 2 U *Taq* polymerase (Takara, Japan). The reactions were first denatured (94°C/3 min), then subjected to 24 cycles of 94°C/30 s, 56°C/60 s, 72°C/60 s, after which a final extension step (72°C/10 min) was given (Xiong et al., 1999). The pre-amplification product was diluted 1:29 in ddH_2_O to provide the template for the subsequent selective PCR, which included a fluorescently-labeled *Eco*RI primer and a non-labeled *Hpa*II-*Msp*I selective (three selective bases) primer. Primer sequences are given in Supplementary Table S1. The set of primer combinations (PCs) used were *Eco*RI selective primer #2 combined with *Hpa*II/*Msp*I selective primer #5 (abbreviated E2 + HM5), E2 + HM7, E3 + HM3, E4 + HM3, E4 + HM7, E4 + HM8, E6 + HM6, E6 + HM8, E7 + HM1, E7 + HM3, E8 + HM2, and E8 + HM8, and the remaining constituents of the reaction were identical to those used for the pre-amplification reaction. The reactions were first denatured (95°C/3 min), then subjected to 13 cycles of 94°C/30 s, 65°C/30 s (reduced by 0.7°C per cycle per cycles), 72°C/30 s, then to 25 cycles of 94°C/30 s, 55°C/30 s, 72°C/ 30 s, and finished by a final extension step of 72°C/7 min. The reaction products were denaturized by heating at 98°C for 3 min followed by cooling on ice and separated using an ABI3730xl (Applied Biosystems, Foster City, CA, USA) device, following the manufacturer’s instructions. Individual fragment sizes were estimated from the migration of the GeneScan LIZ500 size standards, as determined by GeneMapper^®^ v3.7 software (Applied Biosystems). Fragments in the size range 120–480 bp were scored. Two replicate reactions per entry were run, and only reproducible fragments were retained. The statistical test used to interpret variation in MSAP fingerprint followed the suggestion made in (Wang et al., 2013a, 2014a,b):

$$\begin{array}{c} p=\frac{y1+y2}{n1+n2};\;q=1-p;\;\\ \delta_{p1-p2}=\sqrt{pq\left(\frac{1}{n1}+\frac{1}{n2}\right)};\;U=\frac{p1-p2}{\delta_{p1-p2}} \end{array}$$

Where, n1 represents the total sites for a given sample; n2 represents the total sites the mid-values; y1 represents the total DNA methylation sites, hemimethylation sites or fully methylation sites for a given sample, y2 represents the total DNA methylation sites, hemimethylation sites, or fully methylation sites of the mid-values. *p*1 is the percentage of total methylation sites, hemimethylation sites or fully methylation sites for a given sample; *p*2 is the percentage of total methylation sites, hemimethylation sites or fully methylation sites of the mid-values. The Pearson’s *R*^2^ coefficient between the ploidy level and relative *C*-methylation were evaluated using SPSS v19 software.

### The Isolation of CnMET1 and CnDDM1 and Measurement of Transcript Abundance

Total RNA Isolation System (Takara) was used to isolate RNA from fully expanded *C. nankingense* leaves, following the manufacturer’s instructions. A 1 μL aliquot of the resulting RNA (containing about 600 ng) provided the template for the synthesis of the first cDNA strand by SuperScriptIII Reverse Transcriptase (Takara) with random hexamer primers. The subsequent PCR used two degenerate primer pairs (DP1/DP2 and DP3/DP4: sequences given in Supplementary Table S2) which targeted *MET1*, designed from an alignment of the MET polypeptides of *Medicago truncatula* (XP_003619753.1), *Hieracium pilosella* (ACX83570.1), *Nicotiana tabacum* (BAF36443.1), *Prunus persica* (AAM96952.1), *Elaeis guineensis* (ABW96888.1), and *Arabidopsis thaliana* (NP_199727.1). To obtain the full length cDNA prior to a RACE PCR, the sequences were first validated by amplification with a pair of gene-specific primers (SP-F/SP-R: sequences given in Supplementary Table S2; **Supplementary Figure S1**). For the 3′ RACE, the first cDNA strand was synthesized using an oligo (dT) primer incorporating the sequence of the adaptor primer, followed by a nested PCR using the gene-specific primer pair GSP3-1/3-2/3-3 and the adaptor primer (sequences given in Supplementary Table S2). For the 5′ RACE, the nested PCR used the 5′ RACE adaptor primer (Abridged Anchor Primer, AAP), the Abridged Universal Amplification Primer (AUAP) provided with the 5′ RACE System kit v2.0 (Takara) and the internal gene-specific primer pair (GSP5-1/5-2/5-3, sequences given in Supplementary Table S2). The gene’s open reading frame (ORF) was identified using www.ncbi.nlm.nih.gov/gorf/gorf.html and amplified using primers Full-F/Full-R (sequences given in Supplementary Table S2). A multiple sequence alignment of the predicted gene product with homologs present in *M. truncatula, H. pilosella, N. tabacum, P. persica, E. guineensis, A. thaliana, Daucus carota* (AAC39355.1), *H. piloselloides* (ACX83569.1), *N. sylvestris* (CAQ18900.1), *Oryza sativa* (BAG15930.1), *Pisum sativum* (AAC49931.1), *Populus trichocarpa* (XP_002305346.1), *Ricinus communis* (XP_002518029.1), *Solanum lycopersicum* (NP_001234748.1), and *Zea mays* (NP_001105186.1) was carried out using DNAMAN software v5.2.2 (Lynnon Biosoft, Canada), and a subsequent phylogenetic analysis was carried out using MEGA 5.0 software^2^. The same strategy was applied for the isolation of *CnDDM1* (primer sequences given in Supplementary Table S2).

Transcription profiling of *MET1* and *DDM1* was based on RNA extracted from fully expanded fourth and the fifth leaves of *C. nankingense* (2x), *C. indicum* (4x), *C. morifolium* (6x), *C. ornatum* (8x), and *C. crassum* (10x) which are species that were intensively investigated in the previous studies. Prior to its reverse transcription, 30 ng RNA was treated with 10 U of RNase-free DNaseI (Takara) at 37°C for 30 min to remove any contaminating genomic DNA. The first cDNA strand was synthesized by SuperScriptIII Reverse Transcriptase (Takara), according to the manufacturer’s instructions. Quantitative real-time PCR (qRT-PCR) was performed using SYBR Premix Ex TaqTMII (Takara). The reactions were first denatured (95°C/2 min), then subjected to 40 cycles of 95°C/30 s, 55°C/30 s, 72°C/30 s. A previous study suggests that when making cross-species comparison of transcript abundance involving different ploidy levels, care needs to be taken in the selection of reference gene(s) (Wang et al., 2014d), especially in *Chrysanthemum* sp. (Wang et al., 2015). Here, *EF1α* (GenBank accession KF305681), *TUB* (KF305685), *ACTIN* (KF305683), and *PP2A* (KF305684) were used as candidate reference genes (Primer sequences were listed in Supplementary Table S2). The selection of a reference gene was based on geNORM (Vandesompele et al., 2002) and NormFinder (Andersen et al., 2004) Software. The former calculates a stability value (*M*) for each gene, with a lowest *M*-value being taken as an indicator of stable transcription. The latter provides a direct measure of the variation using an ANOVA-based model and ranks the candidate genes accordingly. The data were shown as mean ± SE (*n* = three biological replicates). Each qRT-PCR amplicon was cloned using a PMD19 TA cloning kit (Takara) and sequenced for verification.

### Flow Cytometric Acquisition of Nuclear DNA Content

Flow cytometry was used to determine the relative nuclear DNA contents of *C. nankingense, C. indicum, C. morifolium, C. ornatum*, and *C. crassum*. A 200 mg sample of fresh leaf material (three biological replicates per species) was macerated in 2 mL 15 mM Tris-HCl, pH 7.5, 80 mM KCl, 20 mM NaCl, 20 mM Na_2_EDTA, 2% (v/v) β-mercaptoethanol, 0.05% (v/v) Triton X-100 (Liu et al., 2011). The homogenate was passed through a 50 μm nylon mesh and centrifuged (1,500 × *g*, 10 min, 4°C). Prior to flow cytometry, a 200 μL aliquot of 3 U μL^-1^ RNAase, 50 μg mL^-1^ presidiums iodide was added, and the reaction was kept in the dark at 4°C for 30 min. Flow cytometry was affected with a Coulter EpicsxL device (Beckman Coulter, Miami, FL, USA). Nuclear DNA content was estimated with respect to that of the reference species *C. nankingense*. Each measurement was based on the mean of three technical replicates and only those associated with a coefficient of variation <5% were accepted.

## Results

### MSAP Fingerprinting

**FIGURE 1:**
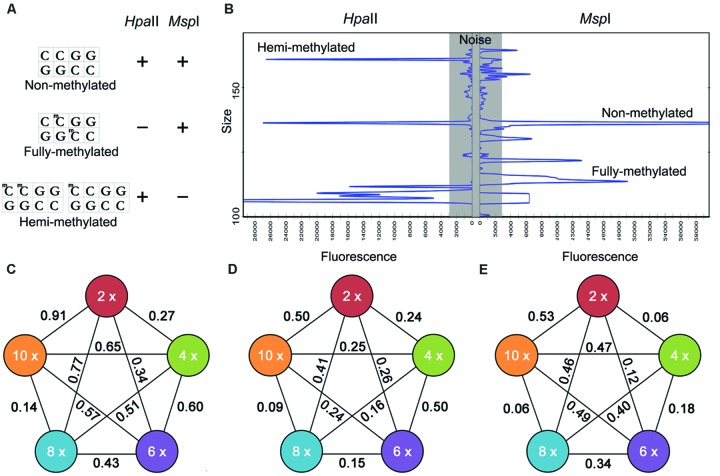
**Representative fragments in methylation sensitive amplification polymorphism (MSAP) profiles **(A,B)** and the relationship between *U-*values and ploidy level (C–E). (A)** *Hpa* II and *Msp* I sensitivities to 5-CCGG methylation status (“+”: enzyme cuts; “-”: enzyme does not cut): three types of fragment generated. Type I: non-methylated, appearing in both the H and M lanes; Type II: fully methylated, present in the M but not the H lanes; Type III: hemi-methylated, present in the H but not the M lanes; **(B)** Examples of MSAP electrophoresis patterns in ABI3730xl; The relationship between *U-*values and ploidy level **(C-E)**. *U-*values associated with **(C)** total methylation, **(D)** full methylation, **(E)** hemi-methylation. A higher *U*-value implies a larger difference between different samples, but only *U*-values>1.96 were statistically significant.

The methylation status of each fragment (**Figures 1A,B**, **Supplementary Data S1**) and the proportion of methylated fragments present in the 20 species is presented in **Table 2**. *A. pacificum* (10x) had the highest proportion of methylated sites (59.2%), followed by *C. ornatum* (8x, 57.1%), *A. shiwogiku* (8x, 56.7%), *A. × marginatum* (8x, 56.6%) and *C. japonense* (6x, 55.6%). The lowest proportions were present in *C. lavandulifolium* (2x, 49.8%), *T. vulgare* (2x, 51.9%) and *C. dichrum* (2x, 52.2%). The range in relative *C*-methylation level was within 10%.

**Table 2:** Variation in *C*-methylation status among the 20 species.

Taxa	Ploidy	Non-methylated sites	Methylated sites		
			Total	Fully methylated	Hemi-methylated
*C. boreale*	2x	47.1%	52.9%	27.5%	25.4%
*C. dichrum*	2x	47.8%	52.2%	26.9%	25.3%
*C. japonicum*	2x	45.4%	54.6%	27.5%	27.1%
*C. lavandulifolium*	2x	50.2%	49.8%	25.4%	24.4%
*C. nankingense*	2x	46.8%	53.2%	27.5%	25.7%
*T. vulgare*	2x	48.1%	51.9%	26.0%	26.0%
*C. chanetii*	4x	44.5%	55.5%	30.2%	25.3%
*C. indicum*	4x	47.6%	52.4%	26.3%	26.1%
*C. japonicum* var. *wakasaense*	4x	45.9%	54.1%	28.2%	25.9%
*C. okiense*	4x	45.6%	54.4%	28.5%	25.9%
*C. yoshinaganthum*	4x	46.7%	53.3%	26.8%	26.5%
*C. japonense*	6x	44.4%	55.6%	28.4%	27.2%
*C. morifolium*	6x	47.5%	52.5%	25.0%	27.5%
*C. vestitum*	6x	45.2%	54.8%	28.8%	26.0%
*C. zawadskii*	6x	45.4%	54.6%	30.0%	24.5%
*A. × marginatum*	8x	43.4%	56.6%	28.1%	28.5%
*A. shiwogiku*	8x	43.3%	56.7%	28.4%	28.3%
*C. ornatum*	8x	42.9%	57.1%	30.0%	27.0%
*C. crassum*	10x	44.1%	55.9%	28.1%	27.8%
*A. pacificum*	10x	40.8%	59.2%	30.5%	28.8%

### Variation in Cytosine Methylation within a Given Ploidy Level

Based on the MSAP profiles, the numbers of non-methylated, hemi-methylated, and fully methylated CCGG sites derived from the MSAP profiles were used to calculate the relative methylation level of the six diploid species; this lay in the range 49.8–54.6% (*U* = 0.11–1.11, *U*_0.05_ = 1.96, a higher *U*-value implies a larger difference between different samples, but only *U*-values >1.96 were statistically significant), broken down into 25.4–27.5% (*U* = 0.05–0.68) fully methylated internal cytosines and 24.4–27.1% (*U* = 0.02–0.69) hemi-methylated external ones. None of the individual values departed statistically from the mid-value of the set of diploid species (**Tables 2** and **3**).

**Table 3:** *U-*values associated with species within a ploidy level.

Taxa	Ploidy	Total methylation	Fully-methylation	Hemi-methylation
*C. boreale*	2x	0.21	0.34	0.10
*C. dichrum*	2x	0.11	0.05	0.18
*C. japonicum*	2x	0.92	0.36	0.69
*C. lavandulifolium*	2x	1.11	0.68	0.59
*C. nankingense*	2x	0.31	0.33	0.02
*T. vulgare*	2x	0.21	0.40	0.16
*C. chanetii*	4x	0.70	1.07	0.31
*C. indicum*	4x	0.69	0.87	0.09
*C. japonicum* var. *wakasaense*	4x	0.07	0.11	0.03
*C. okiense*	4x	0.21	0.25	0.02
*C. yoshinaganthum*	4x	0.29	0.60	0.28
*C. japonense*	6x	0.58	0.18	0.48
*C. morifolium*	6x	0.88	1.62	0.63
*C. vestitum*	6x	0.20	0.37	0.16
*C. zawadskii*	6x	0.09	1.02	0.95
*A. × marginatum*	8x	0.11	0.47	0.35
*A. shiwogiku*	8x	0.16	0.70	0.54
*C. ornatum*	8x	0.03	0.16	0.13
*C. crassum*	10x	1.21	0.95	0.38
*A. pacificum*	10x	1.21	0.95	0.38

For the five tetraploid entries, the proportion of methylated fragments lay between 52.4 and 55.5%. *C. indicum* had the lowest proportion of both total methylated and fully methylated sites, while the lowest proportion of hemi-methylated sites was present in *C. chanetii*. The *U*-values ranged from 0.07 to 0.70 (total methylation), 0.11 to 1.07 (full methylation), and 0.02 to 0.31 (hemi-methylation), and none of the individual values differed significantly from one another (**Tables 2** and **3**).

Among the higher ploidy entries (6–10x), the maximum *U*-values were 1.21 (total methylation), 1.62 (full methylation), and 0.95 (hemi-methylation); these values did not differ significantly from one another (**Tables 2** and **3**). In each species, the hemi-methylated sites were less variable than the fully methylated ones, and the *U-*values associated with the fully methylated sites were greater than those of the hemi-methylated ones in 16 of the 20 species, however, none of these differences were statistically significant (**Table 3**).

### Variation in Cytosine Methylation Status Across Ploidy Levels

Correlation analysis yielded statistically correlations (*R*^2^ = 0.27–0.65) between the ploidy level and relative *C*-methylation. A highest correlation coefficient was obtained in total methylation, while the lowest *R*^2^ was obtained in fully methylation. However, with respect to total methylation level, the *U*-values ranged from 0.14 to 0.91, with the lowest values being associated with the 8 and 10x entries, and the highest with the 2 and 10x entries (**Figure 1C**). With respect to the fully methylated sites, the *U*-values ranged from 0.09 to 0.50, with the lowest associated with the 8 and 10x species and the highest distributed among the 2, 4, 6, and 10x ones (**Figure 1D**). For the hemi-methylated sites, the range in *U*-value was 0.06–0.53, with the lowest recorded in the 8 and 10x species and the highest in the 2, 4, and 10x species (**Figure 1E**). Overall therefore there was no significant difference between ploidy level, either total, full or hemi-methylation levels.

### Isolation of CnMET1 and CnDDM1 Gene and Analysis of its Transcription

**FIGURE 2:**
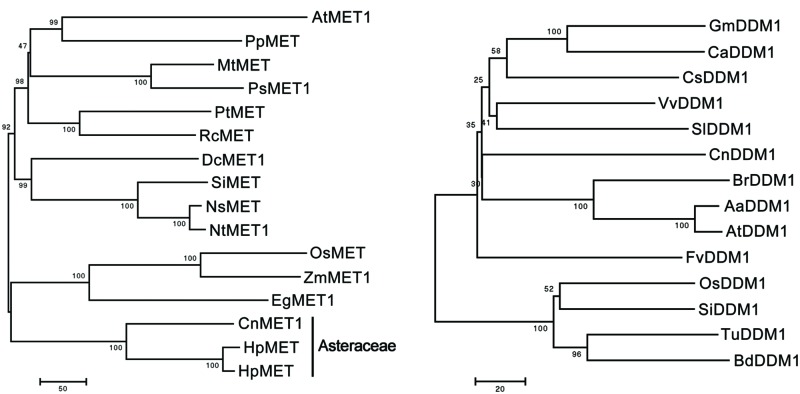
**Phylogeny of MET and DDM1 proteins.** *Arabidopsis thaliana* AtMET1 (NP_199727.1), *Prunus persica* PpMET (AAM96952.1), *Medicago truncatula* MtMET (XP_003619753.1), *Pisum sativum* PsMET (AAC49931.1), *Populus trichocarpa* PtMET (XP_002305346.1), *Ricinus communis* RcMET (XP_002518029.1), *Daucus carota* DcMET1 (AAC39355.1), *Solanum lycopersicum* SlMET (NP_001234748.1), *Nicotiana sylvestris* NsMET (CAQ18900.1), *N. tabacum* NtMET1 (BAF36443.1), *Oryza sativa* OsMET (BAG15930.1), *Zea mays* ZmMET1 (NP_001105186.1), *Elaeis guineensis* EgMET1 (ABW96888.1), *Hieracium pilosella* HpMET (ACX83570.1), *H. piloselloides* HpMET (ACX83569.1), *Vitis vinifera* VvDDM1 (XP_002267239.2), *Glycine max* GmDDM1(XP_003516571.1), *S. lycopersicum* SlDDM1 (XP_004232396.1), *Crocus sativus* CsDDM1 (XP_004149166.1), *B. rapa* BrDDM1 (BAG30707.1), *Cicer arietinum* CaDDM1 (XP_004512037.1), *Fragaria vesca* FvDDM1 (XP_004289144.1), *O. sativa* OsDDM1 (BAF34942.1), *Setaria italica* SiDDM1 (XP_004981943.1), *Triticum urartu* TuDDM1 (EMS59856.1), *Brachypodium distachyon* BdDDM1 (XP_003560489.1), *Arabidopsis arenosa* AaDDM1 (AAP92713.1), *A. thaliana* AtDDM1 (NP_201476.1).

The full length *CnMET1* (Genbank accession KF305682) cDNA was a 5,124 nt sequence, comprising a 4,803 nt ORF, a 92 nt 5′-UTR and a 229 nt 3′-UTR. The sequence showed significant homology to other plant *MET* genes. At the peptide level, the mean level of identity was 71.8%, reaching >80% in the most conserved regions (data not shown). A phylogenetic analysis showed that the most closely related sequences to *CnMET1* were the homologs from *H. pilosella* and *H. piloselloides* (both Asteraceae species); in the conserved regions of the gene, the level of sequence identity was >90% (**Figure 2**). The *CnDDM1* (Genbank KJ560359) sequence encodes a 752 residue product, sharing substantial homology with other plant *DDM* genes (data not shown). Its most closely related sequences were its homologs from *Brassica rapa* and *A. thaliana* (**Figure 2**) and the CnMET1/AtMET1 and CnDDM1/AtMET1 motifs lie parallel to one another and in the most conserved regions. The MET1 cytosine-C5 specific DNA methylase domain and the DDM1 N-terminal domain had an almost identical three-dimensional structure (Data not shown).

**FIGURE 3:**
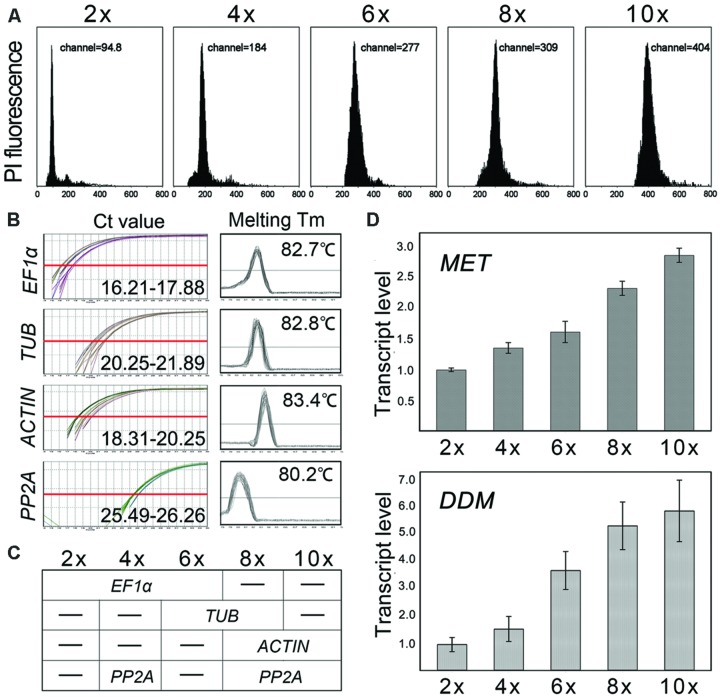
**Nuclear DNA content obtained by flow cytometry and *MET1*/*DDM1* transcription analysis. (A)** Nuclear DNA content of *Chrysanthemum nankingense* (2x), *C. indicum* (4x), *C. morifolium* (6x), *Ajania shiwogiku* (8x) and *C. crassum* (10x); **(B)** The Ct value and melting temperature of each reference genes; the X axis represents the PCR cycle number. The red line represents the threshold fluorescence at which the Ct was determined. None of the reference genes were uniformly transcribed (Ct) in five ploidy levels and species. Melting curve analyses showed that each primer pair amplified a single PCR product. **(C)** Model of reference gene(s) used in five ploidy levels and species; **(D)** Transcript abundance was correlated with ploidy level for both *MET1* and *DDM1*. *PP2A* was selected as inter-run calibrators (IRCs) for *EF1*α, *TUB*, and *ACTIN*, and the coefficient of variation (transcript abundance normalized using *EF1*α_4x-6x_*-TUB*_6x-8x_*-ACTIN*_8x-10x_ vs. using *PP2A*_4x-8x-10x_) < 5%.

Relative to the nuclear DNA content of *C. nankingense*, that of *C. indicum* (4x) was 1.94-fold greater, that of *C. morifolium* (6x) 2.91-fold greater, that of *C. ornatum* (8x) 3.25-fold greater and that of *C. crassum* (10x) 4.25 greater (**Figure 3A**). The relationship between relative nuclear DNA content and ploidy level was not completely linear. This finding is not surprising given the accumulated data across angiosperms which indicate genome down-sizing (due to equilibration or/and deletion of DNA) following hybridization or/and polyploidization.

Cross species comparison of gene expression can be taken with the right reference genes (Wang et al., 2014d). Across all templates, *EF-1a* was the most abundantly transcribed gene, accompanied by the lowest Ct (16.21–17.88), followed by *ACTIN* (18.31–20.25) and *TUB* (20.25–21.89), while *PP2A* (25.49–26.26) was the least abundantly transcribed gene (**Figure 3B**). Melting curve analyses showed that each primer pair amplified a single PCR product (**Figure 3B**). However, none of the reference genes were uniformly transcribed in five ploidy levels and species. Therefore, it was necessary to evaluate the reference genes for normalization in the tested samples. Here, two algorithms, geNorm and NormFinder, were used to determine which of the reference genes would be most suitable in each group. The results shown *EF1α*/*TUBULIN*/*ACTIN*/*PP2A* were predicted to deliver the most reliable level of normalization for 2x vs. 4x vs. 6x/6x vs. 8x/8x vs. 10x/4x vs. 8x vs. 10x ploidy (**Supplementary Figure S2**; **Supplementary Data S2**), as the model shown in **Figure 3C**.

*MET1* was transcribed in all five species, and all the amplicon sequences recovered after qRT-PCR shared the same sequence (data not shown). However, there were substantial inter-specific differences in transcript abundance, in general increasing with the ploidy level. Thus, *MET1* transcript abundance in *C. crassum* (10x) was 1.23-fold that in *C. ornatum* (8x), 1.77-fold that in *C. morifolium* (6x), 2.11-fold that in *C. indicum* (4x) and 2.87-fold that in the *C. nankingense* (2x; **Figure 3D**). Similarly, for *DDM1*, the abundance of transcript in *C. crassum, C. ornatum, C. morifolium*, and *C. indicum* was, respectively, 6.19, 5.62, 3.88, and 1.60-fold that in *C. nankingense* (**Figure 3D**). The results showed that transcript abundance of two genes was increased with genome size, but only *MET1* positively correlated with the nuclear DNA content (*r* = 0.765, *P*= 0.001), while *DDM1* transcript abundance was not correlated (*P*> 0.005).

## Discussion

The Asteraceae represent a relatively young family which has diversified substantially over the past 40 million years. At least three WGD events have occurred during the evolution of the family (Barker et al., 2008; Malinska et al., 2010), and many of its currently extant species are polyploid (Martin et al., 2011). The frequency of polyploidy in the *Chrysanthemum* genus suggests that these species still retain the potential to evolve rapidly (Yang et al., 2006; Liu et al., 2012). MSAP is based on the AFLP technology and can be used effectively to detect polymorphism in DNA methylation patterns within and among different species with no requirement for prior genome information other than the approximate genome size. We therefore consider MSAP to be a suitable technique to evaluate epigenetic changes at the level of DNA methylation in present studies (Wang et al., 2013a, 2014a,b).

The MSAP profiles of the various *Chrysanthemum* species and their close relatives have demonstrated numerous *C*-methylation polymorphisms. *C*-methylation in coding and promoter sequence can have a profound effect on a gene’s expression, so some of these epigenetic polymorphisms may have an impact on the phenotype of plant (Shaked et al., 2001; Adams and Wendel, 2005). *C*-methylation levels and patterns were variable between species sharing the same ploidy level, although the extent of this variation was not that great. Although the extent of *C*-methylation suggested there were little correlations with ploidy level (*R*^2^ = 0.27–0.65), the range in relative *C*-methylation level was within 10% and there was no significant difference between ploidy level, either total, full, or hemi-methylation levels (**Table 2**, **Figure 1**), for example, in the 10*x* species *C. crassum*, 55.9% of the MSAP fragments were methylated, while in the 8*x* species *C. ornatum* the proportion was 57.1%, in the 6*x* species *C. morifolium* 52.5%, in the 4*x* species *C. indicum* 52.4%, and in the 2*x* species *C. japonicum* 54.6%. A similar result has been reported in a number of other genera, which the characteristics of DNA methylation in ployploid may be not consistent with ploidy level (Fortune et al., 2007).

The *MET* sequence is highly conserved across the spectrum from plants to mammals. Its preferred target in plant genomes is the CpG dinucleotide (Lister et al., 2008). In *A. thaliana* at least, the MET1 enzyme acts to maintain the global level of *C*-methylation, since when suppressed via antisense technology, the global level of *C*-methylation, particularly at CpG sites, is strongly reduced, despite the presence of several other methyltransferase genes (Robertson and Jones, 2000; Steward et al., 2000; Kankel et al., 2003; Takeda and Paszkowski, 2006). Here, the sequence of the *Chrysanthemum MET1* homolog is very similar to that of other *MET* genes, as would be expected if MET1 is a functional DNA methyltransferase (**Figure 2**). In contrast to the patterns of *C*-methylation in the *Chrysanthemum* polyploids, *MET1* transcript abundance was positively correlated with genome size. MET1’s primary function is to control *C*-methylation, a form of DNA modification which is necessary to maintain the integrity and stability of the genome. The simplest and most probable explanation for the increased transcript abundance of *MET1* in higher ploidy genomes, in which the level of *C*-methylation is not significantly different from that in low ploidy genomes, is that the quantity of DNA in a high ploidy genome (and therefore the number of CpG dinucleotides) is much higher. Notably, variability of *MET1* transcript abundance was still in a lesser extent (max 2.87-fold). This might be due to MET1-mediated methylation was target-specific and has a high level of methylation efficiency on CG targets (Miki and Shimamoto, 2008; Meyer, 2011).

CpG-methylated sites are targeted by MET1 via its methyl-CpG binding domain, while DDM1 proteins interact with methyl-CpG binding domain proteins and affect their sub-nuclear localization (Zemach et al., 2005). The *A. thaliana DDM1* mutation induces a reduction in DNA methylation level, and is stably inherited (Kakutani et al., 1999). In the present study, the transcript abundances of MET1 and DDM1 genes were all enhanced with increased ploidy level, however, only MET1 positively correlated with the nuclear DNA content, while DDM1 transcript abundance was not correlated. This can be explained by the difference in the DNA methylation efficiency between DDM1 and MET1. Because *MET1* product is DNA methyltransferase, while *DDM1* product functions as a modifier of DNA methyltransferases (Kakutani et al., 1995).

## Conclusion

Inter-specific variability in *C*-methylation (DNA-5′-CCGG sites) within the same ploidy level in the *Chrysanthemum* genus is quite limited, and the nuclear DNA of higher ploidy species is also not necessarily more highly methylated than that of the low ploidy genomes. The transcript abundance of both *MET1* and *DDM1* was enhanced with increased ploidy level but only *MET1* was positively correlated with the nuclear DNA content. Since the higher ploidy genomes harbor a larger number of CpG sites, the enhance of *MET1* transcript abundance would likely have made no significant impact on the overall proportion of methylated sites.

## Author contributions

Conceived and designed the experiments: HW, FC, NT, SC. Performed the experiments: HW, FC, ZG. Analyzed the data: HW, XQ. Contributed reagents/materials/analysis tools: WF, ZG. Wrote the paper: HW, SC. All authors read and approved the final manuscript.

## Conflict of Interest Statement

The authors declare that the research was conducted in the absence of any commercial or financial relationships that could be construed as a potential conflict of interest.

## Footnotes

1 http://rebase.neb.com

2 http://www.megasoftware.net/mega.php

## Supplementary Material

Figure S1 **The strategy for isolating *CnMET1***.

Figure S2 **Average expression stability (*M*) values of the reference genes, as calculated by geNorm, where a low M reflects stability and a high M instability, asterisk is the selected reference gene.(A)** Reference gene selection in 2x vs. 4x/ 4x vs. 6x/6x vs. 8x/8x vs. 10x, *EF1*α, *TUB*, and *ACTIN* were selected; **(B)** As *EF1*α was predicted to deliver the most reliable level of normalization for 2x vs. 4x vs. 6x, *PP2A* has the lowest *M*-value in 4x vs. 8x vs. 10x ploidy, *PP2A* was selected as inter-run calibrators (IRCs) for normalizing *EF1*α, *TUB*, and *ACTIN*.

Data S1 **Binary allele calls for MSAP data**.

Data S2 **Expression stability of the reference genes, as calculated by NormFinder**.
