# Supplementary figures and images for: Limited DNA methylation variation and the transcription of MET1 and DDM1 in the genus Chrysanthemum (Asteraceae): following the track of polyploidy
Source: Front Plant Sci. 2015 Aug 27;6:668. doi: 10.3389/fpls.2015.00668 (PMC4550781; doi:10.3389/fpls.2015.00668)

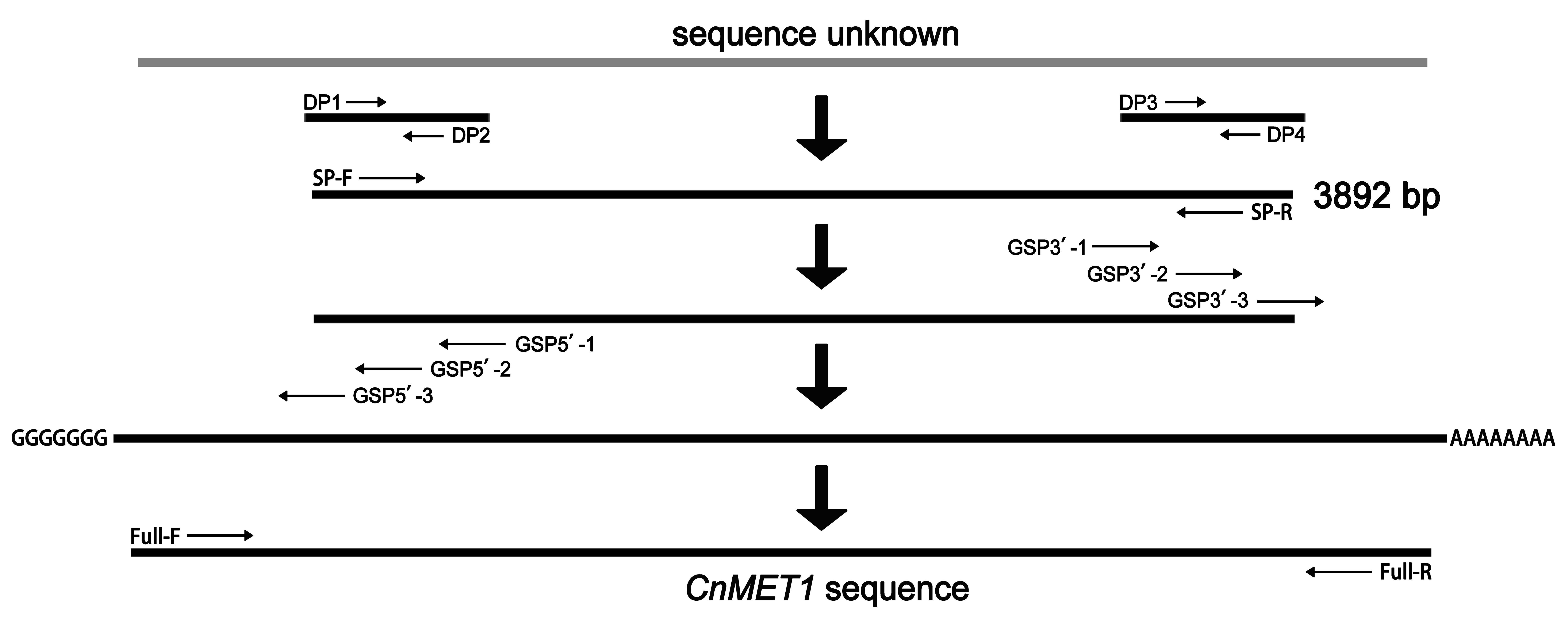

Supplement: Figure S1 — The strategy for isolating CnMET1. [file Image_1.TIF]

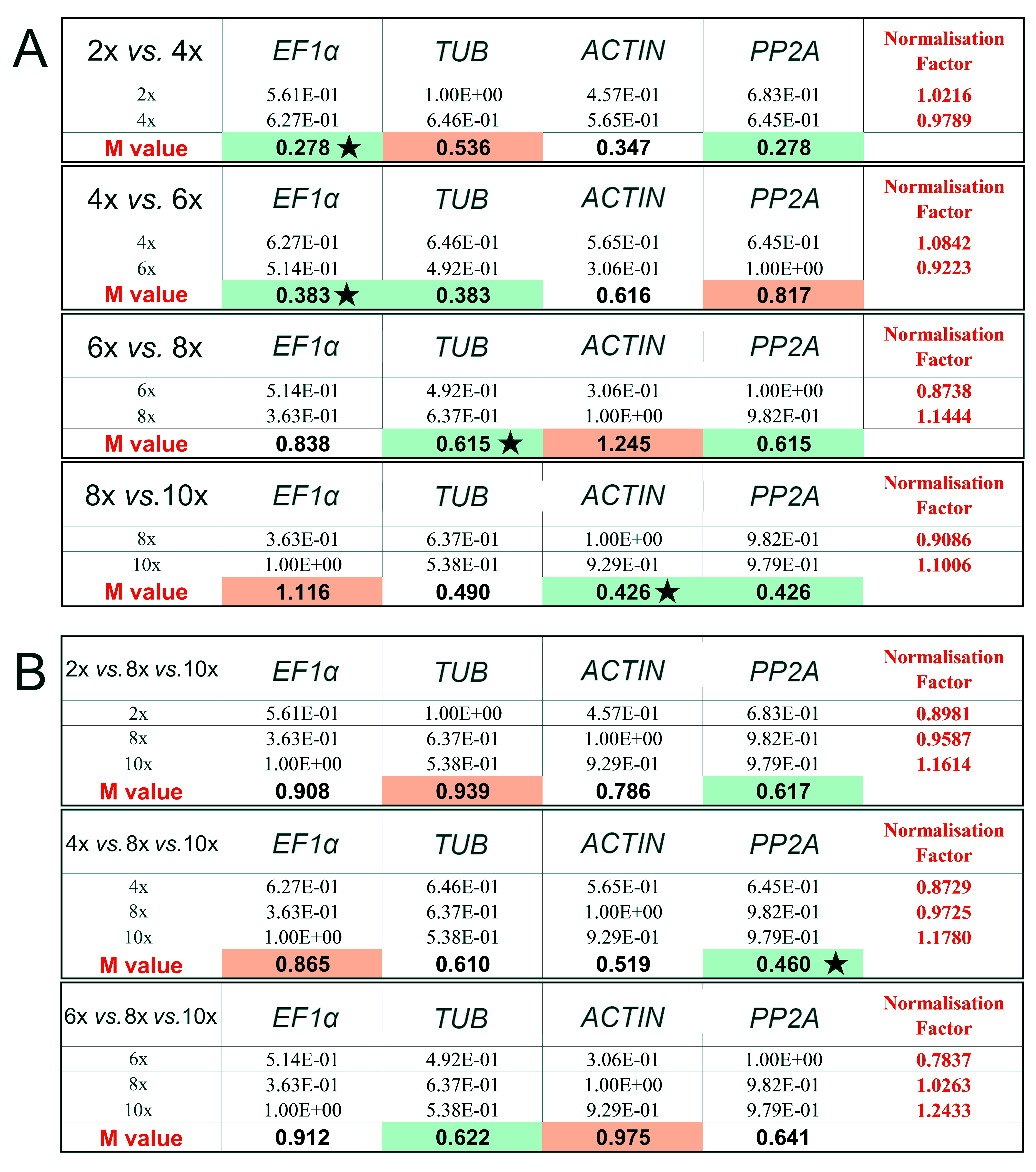

Supplement: Figure S2 — Average expression stability (M) values of the reference genes, as calculated by geNorm, where a low M reflects stability and a high M instability, asterisk is the selected reference gene.(A) Reference gene selection in 2x vs. 4x/ 4x vs. 6x/6x vs. 8x/8x vs. 10x, EF1α, TUB, and ACTIN were selected; (B) As EF1α was predicted to deliver the most reliable level of normalization for 2x vs. 4x vs. 6x, PP2A has the lowest M-value in 4x vs. 8x vs. 10x ploidy, PP2A was selected as inter-run calibrators (IRCs) for normalizing EF1α, TUB, and ACTIN. [file Image_2.TIF]
