# Supplementary material for: Limited DNA methylation variation and the transcription of MET1 and DDM1 in the genus Chrysanthemum (Asteraceae): following the track of polyploidy
Source: Front Plant Sci. 2015 Aug 27;6:668. doi: 10.3389/fpls.2015.00668 (PMC4550781; doi:10.3389/fpls.2015.00668)
Supplement: Supplementary file 5 [file Table_1.DOCX]

**Table S1. Adaptor and primer sequences used for MSAP analysis.**

| **Adaptors/primers** | **Sequence (5**'**–3**'**)** |
| --- | --- |
| ***EcoR*I adaptor-1** | CTCGTAGACTGCGTACC |
| ***EcoR*I adaptor-2** | AATTGGTACGCAGTCTAC |
| ***Hpa*II/*Msp*I adaptor-1** | GATCATGAGTCCTGCT |
| ***Hpa*II/*Msp*I adaptor-2** | CGAGCAGGACTCATGA |
| ***EcoR*I pre-selective primer** | GACTGCGTACCAATTCA |
| ***Hpa*II/*Msp*I pre-selective primer** | ATCATGAGTCCTGCTCGG |
| ***EcoR*I selective primer-2** | GACTGCGTACCAATTCAAG |
| ***EcoR*I selective primer-3** | GACTGCGTACCAATTCACA |
| ***EcoR*I selective primer-4** | GACTGCGTACCAATTCACT |
| ***EcoR*I selective primer-6** | GACTGCGTACCAATTCACG |
| ***EcoR*I selective primer-7** | GACTGCGTACCAATTCAGC |
| ***EcoR*I selective primer-8** | GACTGCGTACCAATTCAGG |
| ***Hpa*II/*Msp*I selective primer-1** | ATCATGAGTCCTGCTCGGTAA |
| ***Hpa*II/*Msp*I selective primer-2** | ATCATGAGTCCTGCTCGGTCC |
| ***Hpa*II/*Msp*I selective primer-3** | ATCATGAGTCCTGCTCGGTTC |
| ***Hpa*II/*Msp*I selective primer-6** | ATCATGAGTCCTGCTCGGTAG |
| ***Hpa*II/*Msp*I selective primer-7** | ATCATGAGTCCTGCTCGGTTG |
| ***Hpa*II/*Msp*I selective primer-8** | ATCATGAGTCCTGCTCGGTCA |
