# Supplementary material for: Limited DNA methylation variation and the transcription of MET1 and DDM1 in the genus Chrysanthemum (Asteraceae): following the track of polyploidy
Source: Front Plant Sci. 2015 Aug 27;6:668. doi: 10.3389/fpls.2015.00668 (PMC4550781; doi:10.3389/fpls.2015.00668)
Supplement: Supplementary file 6 [file Table_2.DOCX]

**Table S2. Primer sequences used for *CnMET1* and *CnDDM1* cloning.**

| **primers** | **Sequence (5'–3')** |
| --- | --- |
| **MET-DP1** | GGATCTCCACCGAGATGGCNGAYTAYGANT |
| **MET-DP2** | CGTCCAGGCCGACCARYTGNTYRWA |
| **MET-DP3** | CCCGGAAGCGGGCNTTYATHTG |
| **MET-DP4** | GGGGGCACGGCGTTNCCDATYTG |
| **DDM1-DP1** | CTGACTGGTGGRCAGCTGRA |
| **DDM1-DP2** | CACCACAYGCTCTAGCSTCA |
| **MET-SP-F** | AACGGCTGTCAAAATCTGCT |
| **MET-SP-R** | TTGTAGCCATCAGGGAAACC |
| **dT-AP** | AAGCAGTGGTATCAACGCAGAGTACTTTTTTTTTTTTTTTT |
| **AP** | AAGCAGTGGTATCAACGCAGAGTAC |
| **MET-GSP3-1** | AGTGGATTTCATCAATGGTGGC |
| **MET-GSP3-2** | GGTTTGGAATACTGGAGGCTGG |
| **MET-GSP3-3** | CCCGGAAGCGGGCNTTYATHTG |
| **DDM-GSP3-1** | GTTATTGGAAAGATTGCTGAA |
| **DDM-GSP3-2** | CGCAATCACAAAGTTCTAAT |
| **DDM-GSP3-3** | TGGACTCGGAATCAATCTCA |
| **MET-GSP5-1** | TTGTAGAGTGACCAGTTGTGAAGC |
| **MET-GSP5-2** | GGAGCGTGTCTTTGCCTTTTATTC |
| **MET-GSP5-3** | GCCTCCAGCAGATTTTGACAGCCG |
| **DDM-GSP5-1** | GCATCATTCATCGCAACTTCGT |
| **DDM-GSP5-2** | TCCTTATTTCATCCCTTTCG |
| **DDM-GSP5-3** | GGTAGACAACGGGGCAATAA |
| **MET-Full-F** | ACAGCGAGTTTGTTAGAATCAGAAGA |
| **MET-Full-R** | TCATAAAGCACAAGGCTACAAAAC |
| **DDM-Full-F** | TAACCTACCCACCACATTTTCCT |
| **DDM-Full-R** | TCACCACAAAACAAGTCACCATTC |
| ***EF1α*-F** | TTTTGGTATCTGGTCCTGGAG |
| ***EF1α*-R** | CCATTCAAGCGACAGACTCA |
| ***PP2A*-F** | GCTTTCGTAATCGCTTTTGG |
| ***PP2A*-R** | ATGGATTCACCTCGATTTGC |
| ***ACTIN*-F** | AGCTTGCATATGTTGCTCTTGA |
| ***ACTIN*-R** | TTACCGTAAAGGTCCTTCCTGA |
| ***TUBULIN*-F** | CTCGAACGCATCAACGTCTA |
| ***TUBULIN*-R** | CGAATCAATAAGCTCGGCTC |
| ***MET*-qRT-PCR-F** | AGGTTGGGATGTGCTTTCAC |
| ***MET*-qRT-PCR-R** | TTCAACCATGCTCTATGTTTCG |
| ***DDM*-qRT-PCR-F** | TGAGGCTAGAGCATGTGGTG |
| ***DDM*-qRT-PCR-R** | CCAGGACCTTTAAGGGGAAG |
